# High-Quality Draft Genome Sequences of Two *Xanthomonas* Pathotype Strains Infecting Aroid Plants

**DOI:** 10.1128/genomeA.00902-16

**Published:** 2016-09-01

**Authors:** I. Robène, S. Bolot, O. Pruvost, M. Arlat, L. D. Noël, S. Carrère, M.-A. Jacques, R. Koebnik, L. Gagnevin

**Affiliations:** a CIRAD, UMR CIRAD-UR Peuplements Végétaux et Bioagresseurs en Milieu Tropical (PVBMT), Saint-Pierre, La Réunion, France; b INRA, Laboratoire des Interactions Plantes Micro-Organismes (LIPM), UMR 441, Castanet-Tolosan, France; c CNRS, Laboratoire des Interactions Plantes Micro-Organismes (LIPM), UMR 2594, Castanet-Tolosan, France; d Université Paul Sabatier, Toulouse, France; e INRA, Institut de Recherche en Horticulture et Semences (IRHS), UMR 1345 SFR 4207 QUASAV, Beaucouzé, France; f IRD, UMR IRD-CIRAD-UM2 Interactions Plantes-Microorganismes-Environnement (IPME), Montpellier, France; g CIRAD, UMR IRD-CIRAD-UM2 Interactions Plantes-Microorganismes-Environnement (IPME), Montpellier, France

## Abstract

We present here the draft genome sequences of bacterial pathogens of the *Araceae* family, *Xanthomonas axonopodis* pv. dieffenbachiae LMG 695 and *Xanthomonas campestris* pv. syngonii LMG 9055, differing in host range. A comparison between genome sequences will help understand the mechanisms involved in tissue specificity and adaptation to host plants.

## GENOME ANNOUNCEMENT

Among the different members of the aroids, the tropical flower anthurium (*Anthurium andreanum* Linden ex André) is an economically important crop cultivated in tropical and temperate areas. Production of anthurium in the world is threatened by *Anthurium* bacterial blight (ABB), which is caused by *Xanthomonas axonopodis* pv. dieffenbachiae (1). Strains of *X. axonopodis* pv. dieffenbachiae isolated from anthurium can infect other aroids, and particularly different ornemental species of *Dieffenbachia*, *Caladium*, *Philodendron*, *Syngonium*, or edible aroids, such as *Colocasia* species. Other *Xanthomonas* strains, first classified as *Xanthomonas campestris* pv. syngonii (2), have a narrower host range and infect *Syngonium* plants causing the bacterial leaf blight of *Syngonium*. Based on rep-PCR and multilocus sequence analysis (MLSA), the two strains LMG 695 and LMG 9055 grouped in the *X. axonopodis* 9.4 cluster (3, 4). Hybridization values (<70%) between LMG 695 or LMG 9055 and the type strain of *X. axonopodis* LMG 982 did not support their inclusion in *X. axonopodis* (5). A polyphasic taxonomic approach based on MLSA, DNA/DNA hybridization, average nucleotide identity (ANI) values, and phenotypic analyses confirmed these results and proposed to reclassify this cluster as *Xanthomonas phaseoli* (6). In addition to differences in pathogenicity, these data also supported the separation of the two categories of strains represented by LMG 695 and LMG 9055 in distinct pathovars named dieffenbachiae and syngonii, respectively.

The genomes of both strains were sequenced using the Illumina HiSeq 2000 platform (GATC Biotech, Germany). Shotgun sequencing yielded 25,320,564 read pairs (12,846,409 100-bp paired-end reads with an insert size of 250 bp, and 12,474,156 50-bp mate-pair reads with an insert size of 3 kb) and 30,521,960 read pairs (17,529,832 100-bp paired-end reads with an insert size of 250 bp and 12,992,128 50-bp mate-pair reads with an insert size of 3 kb) for LMG 695 and LMG 9055, respectively.

A combination of Velvet (7), SOAP*denovo*, and SOAP GapCloser (8) yielded 10 contigs >500 bp (*N*_50_, 1,343,781 bp), with the largest contig being 1,543,007 bp, for a total assembly size of 5,035,943 bp for strain LMG 695, and 33 contigs >500 bp (*N*_50_, 414,753 bp), with the largest contig being 751,399 bp, for a total assembly size of 5,000,894 bp for strain LMG 9055. Genomic contigs were annotated using the EuGene-P annotation pipeline to identify RNAs and protein-coding genes (9). The draft genomes were predicted to contain 4,423 and 4,665 coding sequences (CDSs) for strains LMG 695 and LMG 9055, respectively.

The two genomes have an average nucleotide identity (ANI) of 97.33% (10). The two genomes share >3,700 CDSs, and each contains approximately 200 CDSs, with no orthologs in other *Xanthomonas* species (11). Nineteen and 22 predicted type 3 effector (T3E) genes are present in the LMG 695 and LMG 9055 genomes, respectively. At least 13 of these T3E genes are shared by the two strains.

### Accession number(s).

These whole-genome shotgun projects have been deposited in GenBank under the accession no. CP014347 for strain LMG 695 and LSLD00000000 for strain LMG 9055. The versions described in this paper are the first versions.
